# Are transmembrane 6 superfamily member 2 gene polymorphisms associated with steatohepatitis after pancreaticoduodenectomy?

**DOI:** 10.1002/jgh3.13113

**Published:** 2024-06-25

**Authors:** Tomotaka Mori, Eisuke Ozawa, Ryu Sasaki, Akane Shimakura, Kosuke Takahashi, Yoko Kido, Yasuko Kanda, Satoshi Matsuo, Kazuaki Tajima, Asami Beppu, Yasuhiko Nakao, Masanori Fukushima, Masafumi Haraguchi, Satoshi Miuma, Hisamitsu Miyaaki, Tomohiko Adachi, Susumu Eguchi, Shinji Okano, Kazuhiko Nakao

**Affiliations:** ^1^ Department of Gastroenterology and Hepatology Nagasaki University Graduate School of Biomedical Sciences Nagasaki Japan; ^2^ Department of Gastroenterology and Hepatology Japanese Red Cross Nagasaki Genbaku Hospital Nagasaki Japan; ^3^ Department of Surgery Nagasaki University Graduate School of Biomedical Sciences Nagasaki Japan; ^4^ Department of Pathology Nagasaki University Hospital Nagasaki Japan

**Keywords:** hepatic fibrosis indexes, pancreaticoduodenectomy, PNPLA3, steatohepatitis, TM6SF2

## Abstract

**Aim:**

After pancreaticoduodenectomy, 20–40% of patients develop steatotic liver disease (SLD), and steatohepatitis can be a problem. Although patatin‐like phospholipase domain‐containing 3 protein (PNPLA3) and transmembrane 6 superfamily member 2 (TM6SF2) polymorphisms are involved in SLD and steatohepatitis development, whether this is the case after pancreaticoduodenectomy is unclear.

**Methods and Results:**

Forty‐three patients with pancreatic cancer who underwent pancreaticoduodenectomy at our hospital between April 1, 2018, and March 31, 2021, were included. We extracted DNA from noncancerous areas of residual specimens after pancreaticoduodenectomy and determined PNPLA3 and TM6SF2 gene polymorphisms using real‐time polymerase chain reaction. SLD was defined as a liver with an attenuation value of ≤40 HU or a liver‐to‐spleen ratio of ≤0.9 on computed tomography. We defined high hepatic fibrosis indexes (HFI) instead of steatohepatitis as a Fibrosis‐4 index of ≥2.67 or nonalcoholic fatty liver disease fibrosis score of ≥0.675 in patients with SLD. The cumulative incidence of SLD (*P* = 0.299) and high HFI (*P* = 0.987) after pancreaticoduodenectomy were not significantly different between the PNPLA3 homozygous and minor allele groups. The incidences of high HFI at 1 year after pancreaticoduodenectomy were 16.8% and 27.0% in the TM6SF2 major homozygous and minor allele groups, respectively, with a significant difference in the cumulative incidence (*P* = 0.046).

**Conclusion:**

The TM6SF2 minor allele may contribute to steatohepatitis development after pancreaticoduodenectomy.

## Graphical abstract

Forty‐three patients with pancreatic cancer who underwent pancreaticoduodenectomy (PD) were included. we diagnosed steatotic liver disease (SLD) using CT and high hepatic fibrosis indexes (HFI) instead of steatohepatitis in SLD cases. The incidences of high HFI at 1 year after PD were 16.8 % and 27.0 % in the TM6SF2 major homozygous and minor allele groups, respectively, with a significant difference in the cumulative incidence (p = 0.046). The TM6SF2 minor allele may contribute to steatohepatitis development after pancreaticoduodenectomy.

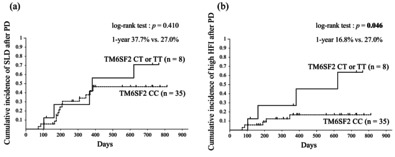

## Introduction

Steatotic liver disease (SLD) occurs at a rate of 20–40% after pancreaticoduodenectomy (PD).^1^, ^2^ The mechanisms underlying SLD after PD might differ from those of metabolic dysfunction‐associated SLD (MASLD). SLD after PD is related to non‐obesity status, malnutrition, and a lack of hyperlipidemia or insulin resistance.^3^ In 2023, the nonalcoholic fatty liver disease (NAFLD) Nomenclature Consensus Group published a multi‐society Delphi consensus statement on new fatty liver disease nomenclature. The name chosen to replace NAFLD was MASLD,^4^ which is defined as a combination of hepatic steatosis and the presence of at least one of five cardiometabolic risk factors.^4^ This revision allows discernment of the underlying factors of SLD with increased precision and eliminates the potential for stigmatization.^4^, ^5^ After changing this nomenclature, the data obtained in NAFLD cases can be used in Japanese patients with NAFLD.^6^, ^7^, ^8^, ^9^, ^10^ Although the mechanism responsible for the development of SLD after PD remains unclear, postoperative malnutrition caused by pancreatic exocrine insufficiency has been proposed as a cause.^11^ The disease is classified into SLD, which rarely progresses, and steatohepatitis, which is progressive and can lead to cirrhosis and hepatocellular carcinoma. There are scattered reports of liver failure due to steatohepatitis as a complication after PD.^12^, ^13^, ^14^ Steatohepatitis also affects the use of adjuvant chemotherapy and is sometimes a serious problem.^15^ The I148M single‐nucleotide polymorphism (rs738409C >G) of patatin‐like phospholipase domain‐containing 3 protein (PNPLA3) was first reported worldwide in 2008 as a susceptibility gene involved in the development of SLD and steatohepatitis.^16^ Exome‐wide association studies in SLD showed that the transmembrane 6 superfamily member 2 (TM6SF2) gene polymorphism (rs58542926) is involved in the development of SLD, steatohepatitis, and liver fibrosis.^17^, ^18^ However, to date, it is not clear whether PNPLA3 and TM6SF2 polymorphisms are involved in the development of SLD and steatohepatitis after PD. Therefore, in this study, we investigated the association of these polymorphisms with SLD and steatohepatitis after PD.

## Methods

### Study design and participants

Patients with pancreatic cancer who underwent PD at our hospital from April 2018 to March 2021 were included. We excluded patients who were not followed up by computed tomography (CT) at 6 months postoperatively and those with preoperative SLD or cirrhosis. Blood work and CT scan data were collected within 1 month preoperatively and at 6, 12, and 24 months postoperatively.

### Measurement of PNPLA3 and TM6SF2 gene polymorphisms

We extracted genomic DNA from formalin‐fixed paraffin‐embedded (FFPE) tissue of noncancerous portions of residual specimens after PD using the QIAamp® DNA FFPE Tissue Kit and Deparaffinization Solution (Qiagen, Venlo, The Netherlands). The SNPs in PNPLA3 and TM6SF2 were genotyped in each sample using the TaqMan SNP genotyping assay kit (Thermo Fischer Scientific, Waltham, MA, USA) containing two allele‐specific TaqMan MGB probes labeled with different fluorochromes and a PCR primer pair, according to the manufacturer's protocol. The following primers were used: PNPLA3 rs738409, AGGCCTTGGTATGTTCCTGCTTCAT[C/G]CCCTTCTACAGTGGCCTTATCCCTC (cat. no. 4351379); TM6SF2 rs58542926, GTGAGGAAGAAGGCAGGCCTGATCT[C/T]GGAGCTGTATTTGCCTTCCATGGTG (cat. no. 4351379).

### Diagnosis of SLD

In most cases, abdominal ultrasonography and magnetic resonance imaging (MRI) are not performed after PD, and SLD is diagnosed using CT. On plain CT, the liver is approximately 50–70 Hounsfield units (HU), and the spleen is approximately 50 HU. In the case of SLD, the CT values of the liver decrease with the degree of fat deposition. Semiquantitative measurement of hepatic fat accumulation is possible by measuring the CT values of the liver and spleen and the liver‐to‐spleen ratio.^19^ We used the average CT values of a circular area of approximately 1 cm^2^ without vascular shadows, which were not affected by artifacts in the transverse sections of the liver and spleen, calculated using the region of interest program, as the respective CT values.^20^ The liver was divided into four sectors (right posterior, right anterior, left medial, and left lateral) using the Couinaud segmentation system. We measured the CT values at four locations in each sector of the liver and two locations in the spleen and calculated the average of each. SLD was defined as a liver CT value of <40 HU or a liver‐to‐spleen CT value ratio of <0.9.^21^, ^22^

### Diagnosis of high hepatic fibrosis indexes

As no pathological examination of the postoperative liver was performed, we decided to diagnose high hepatic fibrosis indexes (HFI) instead of steatohepatitis on the basis of the blood test findings in SLD cases. The American Association for the Study of Liver Diseases Practice Guidance published in 2018 recommends the Fibrosis‐4 (FIB‐4) index and nonalcoholic fatty liver disease fibrosis score (NFS) as less invasive scoring methods.^23^ We defined high HFI as a FIB‐4 index of ≥2.67 or NFS of ≥0.675 in SLD cases.

FIB‐4 index: (age [years] × aspartate aminotransferase (AST) [IU/L])/(platelets [10^9^/L] × √ alanine transaminase (ALT) [IU/L]).

NFS: −1.675 + 0.037 × age (years) + 0.094 × body mass index (BMI) (kg/m^2^) + 1.13 × impaired fasting glucose/diabetes (yes = 1, no = 0) + 0.99 × AST/ALT −0.013 × platelets (×10^9^/L) − 0.66 × albumin (g/dL).

### Statistical analyses

Log‐rank tests were performed to determine whether the incidence of SLD and high HFI after PD differed significantly between the major homozygous PNPLA3 CC group and the PNPLA3 CG or GG groups with the minor allele and between the major homozygous TM6SF2 CC group and the TM6SF2 CT or TT group with the minor allele. We compared each patient's background with the *χ* ^2^ and Mann–Whitney *U* tests. We used the software StatFlex (version 7.0.11; Artech Co., Ltd., Osaka, Japan) for statistical analysis.

### Ethics statements

All researchers involved in this study complied with the “Declaration of Helsinki (revised October 2013)” and the “Ethical Guidelines for Life Sciences and Medical Research Involving Human Subjects” (Ministry of Education, Culture, Sports, Science and Technology, Ministry of Health, Labor and Welfare, Ministry of Economy, Trade and Industry Notification No. 1, 2021). This study was approved by the Nagasaki University Hospital Clinical Research Ethics Committee with permission from the head of the research institution (21 122 001, December 27, 2021). We obtained informed consent in principle from the participants at the time of their outpatient visits. We also obtained consent through an opt‐out procedure from participants who were unable to give direct informed consent.

## Results

### Patient characteristics

**Figure 1:**
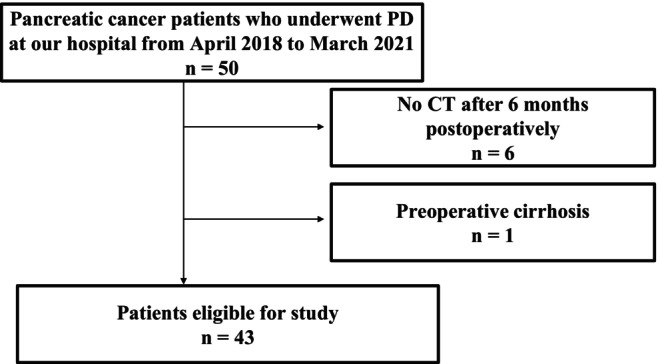
Between April 2018 and March 2021, 50 patients with pancreatic cancer underwent PD at our hospital. Excluding six patients who were not followed up by CT 6 months after surgery and one patient with preoperative cirrhosis, the total number of patients was 43. CT, computed tomography; PD, pancreaticoduodenectomy.

Between April 2018 and March 2021, 50 patients with pancreatic cancer underwent PD at our hospital. Excluding six patients who were not followed up by CT 6 months after surgery and one patient with preoperative cirrhosis, the total number of patients was 43 (Fig. 1). No patient had SLD preoperatively. The patient background characteristics are shown in Table 1.

**Table 1:** Patient characteristics (*n* = 43)

Age, median (range), years	68 (39–83)
Sex, *n* (%)	
Male	21 (48.8)
Female	22 (51.2)
Diabetes, *n* (%)	14 (32.6)
Body mass index, median (range)	20.6 (16.7–34.6)
PNPLA3, *n* (%)	
CC	15 (34.9)
CG	24 (55.8)
GG	4 (9.3)
TM6SF2, *n* (%)	
CC	35 (81.4)
CT	8 (18.6)
TT	0 (0.0)
Platelet counts, median (range), 10^9^/L	229 (96–671)
AST, median (range), U/L	23 (13–110)
ALT, median (range), U/L	20 (9–147)
Albumin, median (range), g/dL	3.9 (1.8–4.9)
Casual blood glucose, median (range), mg/dL	116 (72–214)
HbA1c, median (range), % (NGSP)	6.1 (4.1–12.1)
FIB‐4 index, median (range)	1.42 (0.47–3.82)
NFS, median (range)	−1.26 (−7.45–2.24)
Preoperative chemotherapy, *n* (%)	19 (44.1)
Postoperative chemotherapy, *n* (%)	36 (83.7)
Postoperative pancreatic digestive enzyme replacement drug, *n* (%)	37 (86.0)

FIB‐4 index, Fibrosis‐4 index; HbA1c, Hemoglobin A1c; NFS, nonalcoholic fatty liver disease fibrosis score (NFS); PNPLA3, Patatin‐like phospholipase domain‐containing protein 3; TM6SF2, transmembrane 6 superfamily member 2.

### Cumulative incidence of SLD and high HFI after PD

**Figure 2:**
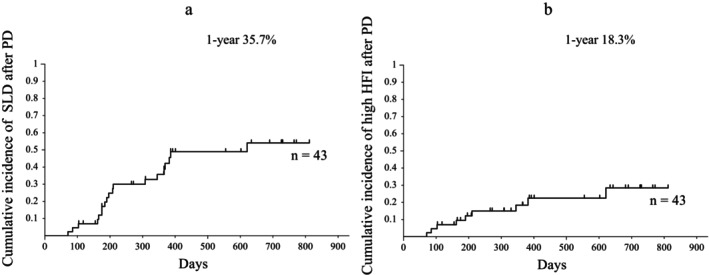
Cumulative incidences of fatty liver (a) and steatohepatitis (b) after PD by according to Kaplan–Meier method analysis. PD, pancreaticoduodenectomy; SLD, Steatotic liver disease; HFI, hepatic fibrosis indexes.

The median observation period was 591 days (range: 85–812 days). Overall, 19 patients (44.1%) developed SLD, and nine (20.9%) developed high HFI. The cumulative SLD and high HFI incidences were 35.7% and 18.3%, respectively, at 1 year (Fig. 2).

### Comparison with and without PNPLA3 minor allele retention

**Figure 3:**
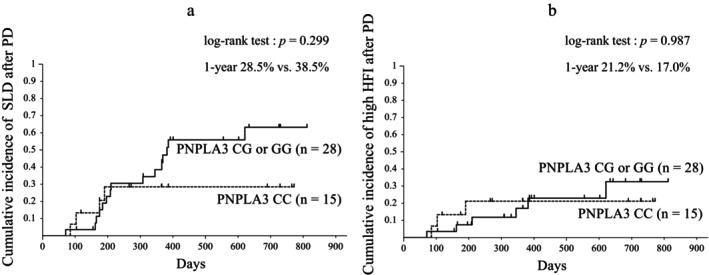
Cumulative incidences of fatty liver (a) and steatohepatitis (b) after PD by according to Kaplan–Meier method analysis in patients with PNPLA3 of CC *versus* CG or GG. PD, pancreaticoduodenectomy; SLD, Steatotic liver disease; HFI, hepatic fibrosis indexes; PNPLA3, Patatin‐like phospholipase domain‐containing protein 3.

The incidences of SLD at 1 year after PD were 28.5% and 38.5% in the PNPLA3 major homozygous and minor allele groups, respectively, with no significant difference in the cumulative incidence (*P* = 0.299) (Fig. 3a). The incidences of high HFI at 1 year after PD were 21.2% and 17.0% in the PNPLA3 major homozygous and minor allele groups, respectively, with no significant difference in the cumulative incidence (*P* = 0.987) (Fig. 3b).

### Comparison with and without TM6SF2 minor allele retention

**Figure 4:**
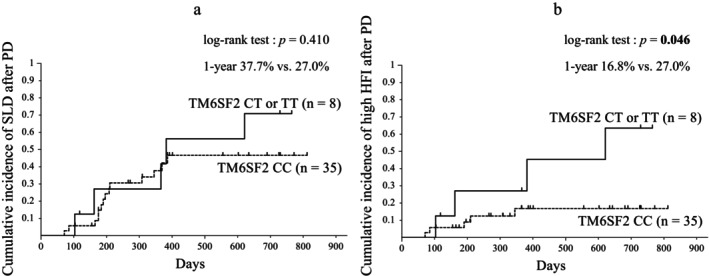
Cumulative incidences of fatty liver (a) and steatohepatitis (b) after PD by according to Kaplan–Meier method analysis in patients with TM6SF2 of CC *versus* CT or TT. Values in bold indicate statistical significance at *P* < 0.05. PD, pancreaticoduodenectomy; SLD, Steatotic liver disease; HFI, hepatic fibrosis indexes; TM6SF2, transmembrane 6 superfamily member 2.

The TM6SF2 minor allele group had a significantly higher preoperative BMI than the major homozygote group (median, 25.3 *vs* 20.2; *P* = 0.010). The other patient background characteristics were not significantly different between the groups (Table 2). The incidences of SLD 1 year after PD were 37.7% and 27.0% in the TM6SF2 major homozygous and minor allele groups, respectively, with no significant difference in the cumulative incidence (*P* = 0.410) (Fig. 4a). The incidences of high HFI at 1 year after PD were 16.8% and 27.0% in the TM6SF2 major homozygous and minor allele groups, respectively, with a significant difference in the cumulative incidence (*P* = 0.046) (Fig. 4b).

**Table 2:** Patient characteristics by TM6SF2 CC and CT or TT

	TM6SF2 CC (*n* = 35)	TM6SF2 CT or TT (*n* = 8)	*P*‐value
Age, median (range), years	69 (39–83)	66 (61–71)	0.730
Sex, *n* (%)			0.456
Male	16 (45.7)	5 (62.5)	
Female	19 (54.3)	3 (37.5)	
Diabetes, *n* (%)	10 (28.6)	4 (50.0)	0.403
Body mass index, median (range)	20.2 (16.7–34.6)	25.3 (20.1–31.5)	**0.010**
PNPLA3, *n* (%)			0.230
CC	11 (31.4)	4 (50.0)	
CG	20 (57.1)	4 (50.0)	
GG	4 (11.4)	0 (0.0)	
Postoperative chemotherapy, *n* (%)	30 (85.7)	6 (75.0)	0.597
Postoperative pancreatic digestive enzyme replacement drug, *n* (%)	30 (85.7)	7 (87.5)	1.000

Values in bold indicate statistical significance at *P* < 0.05.

PNPLA3, Patatin‐like phospholipase domain‐containing protein 3; TM6SF2, transmembrane 6 superfamily member 2.

## Discussion

Reduced pancreatic exocrine capacity and undernutrition are associated with the development of SLD after PD.^11^ Moreover, enhanced fatty acid uptake into hepatocytes, lipogenesis, and disruption of very low‐density lipoprotein excretion into the circulation are possible mechanisms of steatogenesis after PD.^24^ However, the detailed mechanisms remain unclear. We hypothesized that minor allele carriers of PNPLA3 and TM6SF2 might also develop SLD and steatohepatitis after PD, although the mechanism is different from that of typical MASLD.

In a previous report, multivariate logistic regression analysis showed that pancreatic head cancer was associated with an increased risk of developing SLD after PD.^25^, ^26^ Therefore, we included patients with pancreatic cancer who underwent PD to ensure a uniform patient background. It was reported that 21.7% of patients with pancreatic cancer developed SLD after 6 months of PD and 29.8% and 46.2% of patients developed SLD within 12 months of PD.^15^, ^26^, ^27^ The present study's results are also consistent with findings of these previous reports, as 35.7% of patients with pancreatic cancer developed SLD within 12 months of PD. Few studies have reported the incidence of steatohepatitis after PD. To our best knowledge, there are no other reports on the overall incidence of steatohepatitis after PD. In one report, 20 of 54 patients developed SLD within 12 months of PD, and two of four patients who underwent liver biopsy for liver damage had steatohepatitis (i.e., ≥3.7%)^2^; in another report, 14 of 60 patients developed SLD within 12 months of PD, and all eight patients, who underwent liver biopsy with consent, had steatohepatitis (i.e., ≥13.3%).^25^ Although we did not perform a liver biopsy in this study, 18.3% of the patients with pancreatic cancer developed high HFI within 12 months after PD, consistent with findings of previous reports.

PNPLA3 is highly expressed in human hepatic stellate cells and hepatocytes. In both cell types, this protein is located in lipid droplets and has hydrolase activity toward triglycerides (TGs) and retinyl esters in hepatocytes and hepatic stellate cells, respectively. The I148M mutation results in the loss of protein function, with fat retention in hepatocytes and retinol retention in hepatic stellate cells. The molecular mechanism by which PNPLA3 promotes fibrosis by transversing cytosine (C) to guanine (G) remains unknown.^28^ Contrary to our expectations, there was no difference in the incidence of SLD or high HFI after PD between PNPLA3 major homozygous and minor allele groups. This may be partly due to the insufficient number of cases; however, the detailed reason for this is unclear.

In the liver, TM6SF2 can convert zymosterol to 5‐a‐cholesta‐7,24‐dien‐3b‐ol and increase 7‐dehydrocholesterol reductase expression, which promotes cholesterol biosynthesis. TM6SF2 also decreases the expression of ATP‐binding cassette subfamily G member 5 and ATP‐binding cassette subfamily G member 8, which are associated with total cholesterol and low‐density lipoprotein‐cholesterol levels in humans. Additionally, TM6SF2 may enhance the expression of diacylglycerol acyltransferase (DGAT) 1, DGAT2, and Acyl‐CoA synthetase short‐chain family member 2, which are involved in TG synthesis. In the small intestine, TM6SF2 promotes neutral lipid absorption and release, increasing circulating TG levels. It has been suggested that inhibition of TM6SF2 protects against cardiovascular disease, likely at the expense of increasing the risk for MASLD and diabetes.^18^, ^29^, ^30^, ^31^, ^32^ Akuta et al. found that the rates of decreasing triglyceride levels were significantly higher in patients in the TM6SF2 CT group than in those in the CC or TT group.^33^ Additionally, Seko et al. reported that TM6SF2 was significantly associated with the onset of liver‐related event.^34^ In this study, the group with the TM6SF2 minor allele had a significantly higher preoperative BMI and incidence of high HFI. The incidence of SLD after PD has been reported to be associated with a lower postoperative BMI.^15^, ^35^ However, some reports have indicated that the incidence of SLD is significantly higher in the high preoperative BMI group than in the low BMI group,^36^, ^37^ whereas others have indicated that the incidence of SLD is significantly higher in the low preoperative BMI group than in the high BMI group.^25^ The TM6SF2 minor allele group developed significantly higher HFI after PD, as hypothesized, although with a bias of higher preoperative BMI. These results indicated that the TM6SF2 minor allele may be involved in the development of steatohepatitis after PD as well as in typical metabolic dysfunction‐associated steatohepatitis (MASH).

Contrary to our expectation, we could not find any association between the presence of the TM6SF2 minor allele and the development of SLD after PD. Previous studies have shown that hepatic TG accumulation may not be directly hepatotoxic. This was demonstrated in mice by silencing the hepatic gene expression of DGAT2, a key enzyme that mediates the conversion of free fatty acids to TGs.^38^ Rather than ameliorating steatohepatitis, the consequent reduction in hepatocyte TG synthesis was associated with increased fatty acid oxidation, particularly through cytochrome P450 family 2 subfamily E member 1, leading to greater oxidative stress, greater cellular damage, and higher serum transaminase levels.^38^ Thus, one study speculated that the function of TM6SF2 and the mechanism through which TM6SF2 drives MASLD‐associated hepatic fibrosis may be something other than increased TG accumulation.^39^ The etiology and pathogenesis of MASLD and MASH are conventionally known as the two‐hit theory, in which SLD occurs as the first hit and progresses to MASH as the second hit.^40^ However, not all cases of steatohepatitis develop via a SLD, and the concept of the multiple parallel hit hypothesis has been proposed in recent years.^41^, ^42^ The idea is that the various factors involved in the development of hepatic lipidosis, inflammation, and fibrosis act in parallel on the liver to cause steatohepatitis. Nagaya et al. examined changes in gene expression associated with steatohepatitis development in patients after PD compared with healthy individuals and patients with conventional MASH. The results reported that enhanced fatty acid uptake into hepatocytes and lipogenesis, upregulation of peroxisome proliferator‐activated receptor gamma, and disruption of very low‐density lipoprotein excretion into the circulation are possible mechanisms of steatogenesis after PD. Additionally, elevated messenger RNA levels of myeloid differentiation primary response 88 and superoxide dismutases in post‐PD steatohepatitic livers suggest significant activation of the innate immune response and augmentation of oxidative stress generation.^24^ The difference in the mechanism of SLD development after PD, which may be more inflammation‐prone, and conventional MASLD may be due to the difference in how TM6SF2 works, although the exact reason is unclear.

This study has limitations because it was a single‐center, nonrandomized, retrospective study. In addition, we did not use pathology, fibrosis marker measurements, abdominal ultrasonography, or MRI to diagnose SLD or steatohepatitis. There was also a bias in that the TM6SF2 minor allele group had a higher preoperative BMI than the major homozygous group. It was not possible to fit the multivariate Cox proportional hazard modeling analysis including the covariates of TM6SF2 for the development of high HFI because of the insufficient number of high HFI events. Therefore, it is necessary to consider larger multicenter randomized controlled trials, pathological diagnoses, measurement of fibrosis markers, and abdominal ultrasonography and MRI examinations in the future. Despite these limitations, to our best knowledge, this is the first study to evaluate whether TM6SF2 minor allele is a risk factor for post‐PD high HFI. Our findings may lead to future studies on the prevention of post‐PD steatohepatitis.

In conclusion, the presence of the TM6SF2 minor allele may contribute to the development of steatohepatitis after PD.
